# Case report: A case of immune thrombocytopenia combined with Hashimoto's thyroiditis and Helicobacter pylori infection in a child

**DOI:** 10.3389/fped.2023.1169064

**Published:** 2023-06-09

**Authors:** Lihong Yang, Dongqiong Xiao, Xihong Li, Chunqi Lai, Yuhao Chen, Lingli Pan

**Affiliations:** ^1^ Department of Emergency, West China Second University Hospital, Sichuan University, Chengdu, China; ^2^ Key Laboratory of Birth Defects and Related Diseases of Women and Children (Ministry of Education), Sichuan University, Chengdu, China

**Keywords:** children, immune thrombocytopenia, Hashimoto’s thyroiditis, helicobacter pylori, treatment

## Abstract

Immune thrombocytopenia (ITP) is one of the most prevalent acquired bleeding disorders in children, which is primarily characterized by a decrease in platelet count. It can be classified into two subtypes: primary ITP and secondary ITP. The underlying mechanisms causing ITP are complex and not fully comprehended. Helicobacter pylori (H. pylori) infections can lead to ITP and potentially trigger various autoimmune diseases. Furthermore, there is evidence of a correlation between thyroid disease and ITP. In this case report, we describe the case of an 11-year-old patient who presented with ITP, Hashimoto's thyroiditis (HT), and H. pylori infection. Following anti-H. pylori treatment and thyroxine supplementation, the child's platelet count increased compared to the previous count. The limitation of this report is that the platelet count of this child returned to normal after anti-H. pylori and thyroxine supplementation, so we cannot distinguish the effect of anti-H. pylori and thyroxine supplementation on the platelet count in this child. Despite this limitation, we still believe that early screening for thyroid function and H. pylori, as well as prompt eradication of H. pylori, along with thyroxine supplementation, may be beneficial in treating and improving the prognosis of children diagnosed with ITP.

## 1. Introduction

Immune thrombocytopenia (ITP) is a frequent immune-mediated bleeding disorder in children, with an incidence of 5–10 cases per 100,000 children annually (1, 2). Increased destruction of peripheral platelets and/or reduced platelet production underlies the disease's pathophysiology. Although patients usually present with auto-antibodies against specific platelet membrane glycoproteins, the exact trigger for antibody production remains unknown. Furthermore, platelet auto-antibodies can damage bone marrow megakaryocytes, limiting platelet production. Yet, research indicates that most patients do not exhibit a significant decrease in platelet production (1, 3). The infection could be a crucial trigger in the development of platelet autoimmunity in ITP (4). Studies have shown that platelet recovery after Helicobacter pylori (H. pylori) eradication therapy may be due to cross-reactivity between anti-H. pylori antibodies and platelets, which affects physiological pathways such as the balance of regulatory Fc*γ* receptors (5–8). Due to modified immune responses and the production of auto-reactive antibodies, ITP, and other autoimmune diseases frequently coexist. Thyroid disorders are closely associated with ITP, and thyroid disease management has been shown to improve thrombocytopenia, even when ITP treatment is unsuccessful (9, 10).

Herein, we describe a case of a child who was admitted to our hospital with ITP, HT, and H. pylori infection. To aid in comprehensively understanding the study topic, we also incorporate relevant information from literature reviews.

## 2. Case presentation

An 11-year-old boy was admitted to a local hospital with scattered petechiae all over his body. Upon presentation, the child denied experiencing redness, pain, or itching in both lower extremities, fever, fatigue, malaise, nausea, vomiting, dizziness, headache, throat discomfort, abdominal pain, acid reflux, feeling of fullness, constipation, blood in stool or urine, arthralgia and weighed 31 kg. No hepatosplenomegaly was detected, and routine blood tests revealed thrombocytopenia with a minimum platelet count of 5 × 10^9^/L. A bone marrow aspiration was scheduled, which indicated a decrease in platelet-producing megakaryocytes. The child was thus diagnosed with ITP and subjected to hormone shock therapy, which caused their platelet count to rise, reaching a maximum of 44 × 10^9^/L. However, during oral hormone therapy, the platelet count fluctuated around 20 × 10^9^/L, leading to their referral to our hospital due to poor treatment outcomes and lowered platelets. The child denied having a history of vaccination over a month ago, a recent cold, or any other medication, and their family reported no similar history of such a condition. Upon admission, a physical examination revealed a slightly congested pharynx but no bilateral enlargement of the tonsils, thyroid gland enlargement or pressure pain, cardiac, respiratory, gastrointestinal, or neurological abnormalities, scattered petechiae, and a few new bleeding spots on both lower limbs, no skin swelling, and reduced skin temperature. The routine blood tests conducted on the day of admission showed white blood cells of 12.2 × 10^9^/L, red blood cells of 4.35 × 10^12^/L, platelets of 28 × 10^9^/L, and normal results in all other indicators.

**Figure 1:**
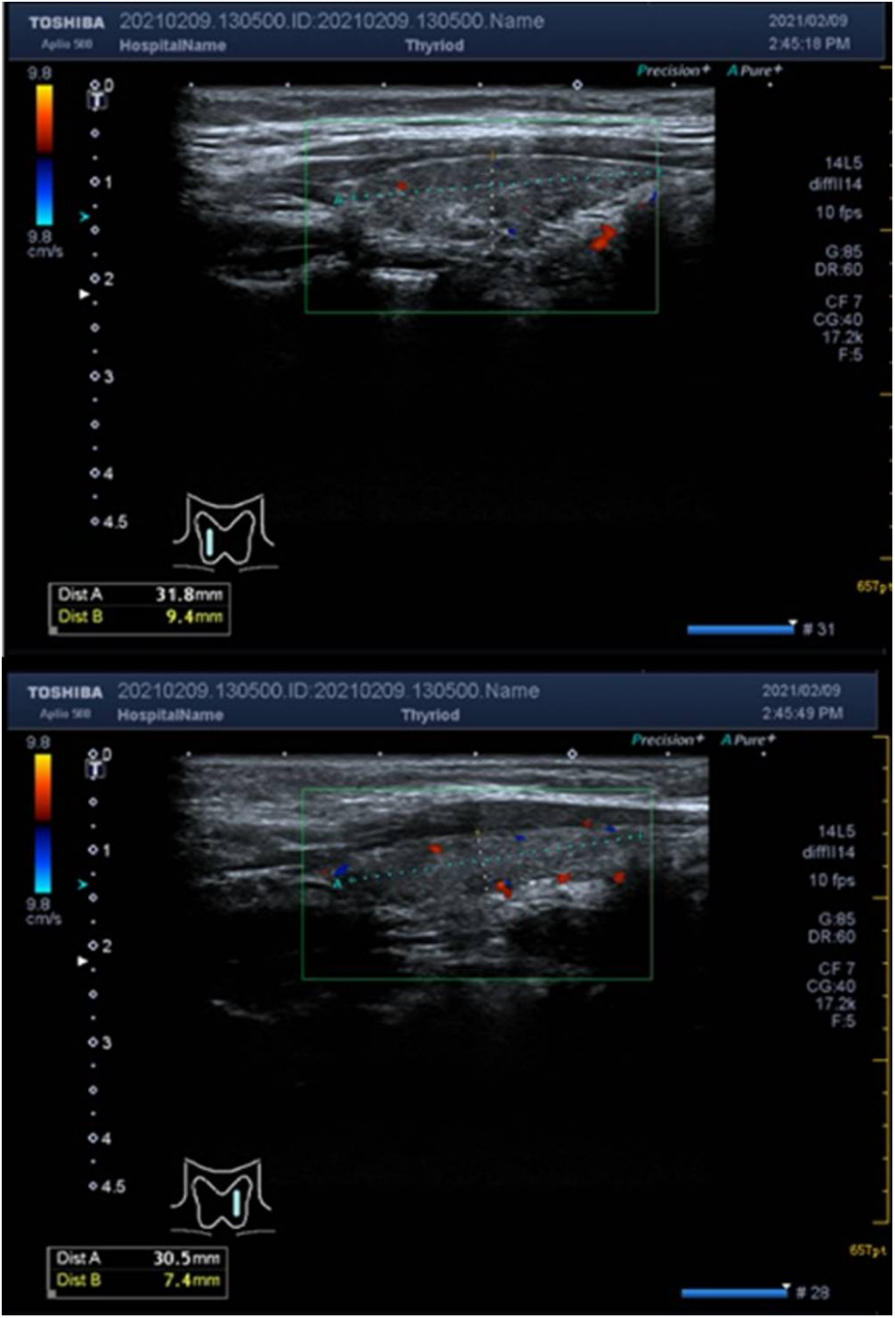
The dashed line above the thyroid ultrasound shows the size of the anterior-posterior and superior-inferior diameters. The anterior-posterior diameter of the right lobe of the thyroid gland was 0.9 cm, the left and right diameters were 1.1 cm, the upper and lower diameters were 3.2 cm, and the anterior-posterior diameter of the left lobe was 0.7 cm, the left and right diameters were 1.0 cm, the upper and lower diameters were 3.1 cm, and the thickness of the isthmus was about 0.14 cm. The parenchymal echogenicity was hypoechoic and inhomogeneous, no exact occupancy was seen, and no significant abnormal blood flow signal was detected.

The child underwent an immunoglobulin shock treatment, resulting in a rise in platelet count to 57 × 10^9^/L on the next day's recheck. However, the treatment did not provide satisfactory results. Multiple tests were conducted to investigate the cause of the low platelet count. Liver and kidney function tests were performed and the results showed no abnormalities. Thyroid function testing revealed that T3 (1.35 nmol/L) and T4 (90.4 nmol/L) levels were within normal limits, but TSH (6.811 mIU/L) levels were elevated, indicating an underactive thyroid. FT3(4.71 pmol/L) levels were lower than the reference range, while FT4(16.16 pmol/L) levels were normal. Antibody testing showed higher TGAB (94.7 U/ml) and TPOAB (848.3 U/ml) levels compared to the reference range, indicating a possible autoimmune disorder (Supplementary Table S1). The child tested positive for H. pylori antibodies, negative for antiplatelet and antinuclear antibodies, and mildly elevated IgE, but no abnormalities were found for IgM, IgG, IgA, or complement. Ultrasound of the thyroid showed hypoechoic and heterogeneous parenchymal echogenicity with no significant abnormal blood flow signal, and the size of the thyroid was consistent with HT (Figure 1).

We found that this child had H. pylori infection in addition to ITP and HT. The child's platelet count repeatedly decreased, which was considered to be related to a previous H. pylori infection, and we empirically selected amoxicillin, clarithromycin, and omeprazole for anti-H. pylori infection treatment.

After nine days of treatment, the child's platelet count improved and increased to 192 × 10^9^/L. We advised the child to continue taking oral thyroxine, methylprednisolone, and anti-H. pylori therapy. However, during a follow-up visit seven days later, we found that the child's platelet count had decreased to 152 × 10^9^/L. Since the child had already completed the full two-week course of anti-H. pylori treatment, we recommended that he discontinue amoxicillin, clarithromycin, and omeprazole. Instead, we advised him to continue with oral thyroxine and methylprednisolone therapy to manage his thyroid condition and ITP respectively.

At the scheduled review one week later, the child did not show any significant bleeding tendency, however, his platelet count had decreased to 92 × 10^9^/L. Further examination of the child's thyroid function revealed that the T3 level was below the reference range at 0.7 nmol/L, while the T4 level was within the normal range at 77.50 nmol/L. The TSH level was within the normal range at 0.758 mIU/L, while FT3 levels and FT4 levels were also within the normal range at 2.90 pmol/L and 17.46 pmol/L, respectively. Both TGAB (67.3 U/ml) and TPOAB (238 U/ml) levels were decreased from the previous test results, but were still above the reference range (Supplementary Table S1). In light of these findings, we recommend implementing an adjusted treatment plan for the child. Specifically, we proposed increasing the dose of methylprednisolone and thyroxine to better control the child's thyroid function and platelet count.

**Figure 2:**
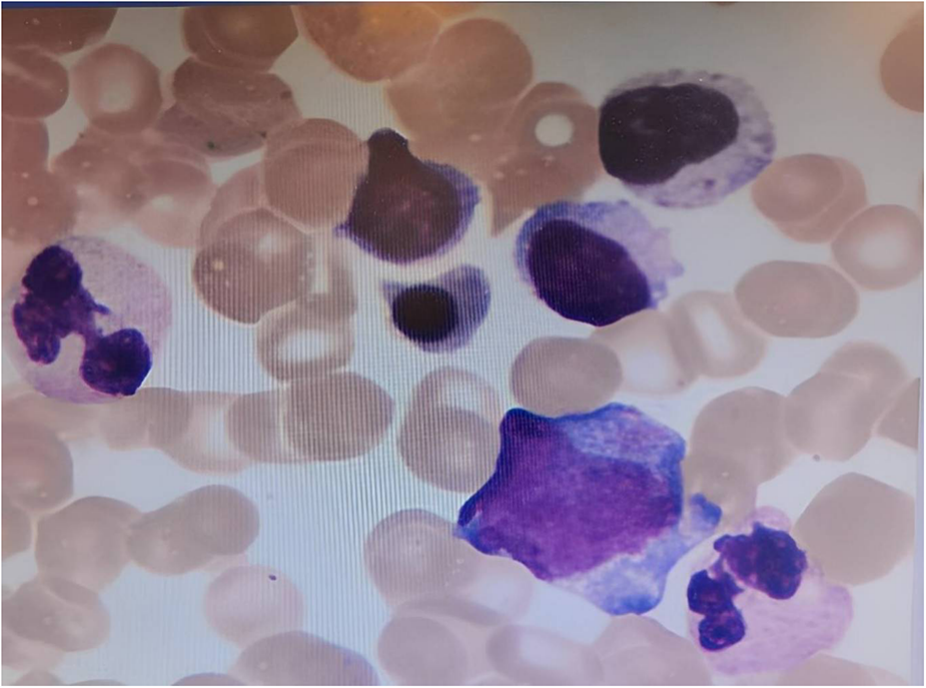
Bone marrow cytology image of the child in this case. Magnification:40, hematoxylin and eosin (H&E) staining. Bone marrow cell smear suggested 98 megakaryocytes and 17/50 platelet-producing megakaryocytes.

One week later, the child's platelet count had further decreased to 44 × 10^9^/L. In response, we scheduled a bone marrow aspiration for the child, and upon examination, found 98 megakaryocytes and 17 out of 50 platelets (Figure 2). In light of these findings, we recommended that the child continue taking oral thyroxine and methylprednisolone, while also starting eltrombopag to help increase his platelet count.

After a week more, the child's platelet count further decreased to 41 × 10^9^/L. We continued to advise the child to take oral medications as previously prescribed, including thyroxine, methylprednisolone, and eltrombopag.

**Figure 3:**
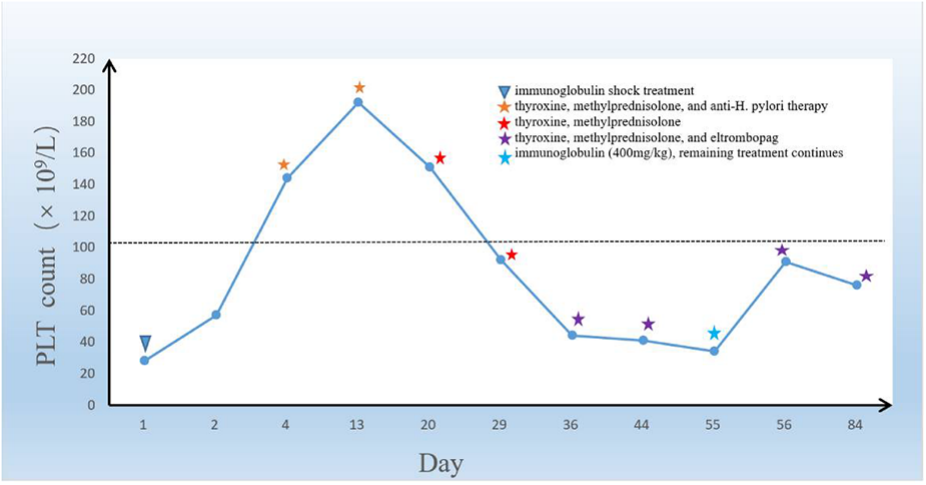
The graph shows the trend of the change in the platelet count of the boy in the case during the treatment period. In the graph above, the child's treatment is marked with an asterisk.

Eleven days later, the child returned to our hospital for a follow-up visit, and the retest of the platelet count showed 34 × 10^9^/L. At this time, the child's mother reported that the child had completed a two-week course of anti-H. pylori treatment and underwent a ^13^C-urea breath test at a local hospital, which suggested positive results four weeks after stopping the medication. Then, we administered another dose of immunoglobulin to the child at a dosage of 400 mg/kg, and their platelet count increased to 91 × 10^9^/L. We instructed the child to continue taking his oral medications, including eltrombopag, thyroxine, and methylprednisolone, without any dose adjustment. A month later, the child returned to our hospital for a re-examination, and their platelet count was maintained at 76 × 10^9^/L with no reported bleeding tendency or complications (Figure 3).

## 3. Discussion

Herein, we present the case of a male child with ITP who presented with HT and previous H. pylori infection. The child was initially treated with immunoglobulins, however, the therapeutic approach was insufficient. After the implementation of anti-H. pylori treatment and thyroxine supplementation, the child's platelet count recovered. A ^13^C-Urea breath test revealed persistent infection after 4 weeks of treatment discontinuation. At this time the child had a reduced platelet count. It suggested that the eradication of H. pylori was incomplete in this child. We speculate that the decrease in the child's platelet count is related to incomplete treatment of the H. pylori infection, but acknowledge that further confirmatory studies are needed to validate this hypothesis.

H. pylori is a pathogenic microorganism known to colonize the stomach and cause various diseases, including gastric disturbances and extra-gastric disorders (11). Typically, H. pylori infection occurs in childhood, and if left untreated, it can persist and lead to lifelong infection of the stomach (12). Despite being associated with gastric inflammation, microscopic or macroscopic, the vast majority of H. pylori-infected children do not experience any symptoms (13). Pathogenic H. pylori are genetically diverse and adapt to survive in the unique microenvironments present in different anatomical parts of the stomachs of individual patients (14). Furthermore, H. pylori bacteriophages also promote the development of a ﬂexible H. pylori community with variable characteristics (15).

Research has shown that H. pylori infection is increasingly associated with the development of ITP (16). H. pylori eradication therapy has also been demonstrated to significantly improve platelet counts in ITP patients, and this intervention has been shown to inhibit the production of antiplatelet antibodies, with varying degrees of ITP remission observed in different patients (17–20). Recent studies have further established the role of H. pylori eradication in improving platelet count recovery in ITP patients, with successful H. pylori eradication shown to increase platelet counts dramatically, whereas persistent infection is associated with poor platelet recovery due to eradication failure (21). The mechanistic pathways by which H. pylori promotes ITP development are postulated to include molecular mimicry, increased plasmacytoid dendritic cell count, and the host immune response to virulence factors such as vacuolating-associated cytotoxin gene A (VacA) and cytotoxin-associated gene A (CagA). The anti-CagA and/or anti-VacA antibodies react to platelets (cross-reaction), leading to platelet aggregation and destruction (22–24). Studies have proposed an alternative pathway for H. pylori induction of chronic ITP, namely the formation of an immune complex consisting of the H. pylori antigen Lpp20, H. pylori-specific antibodies, and platelets (5, 25). These studies have shown that levels of H. pylori-specific antibodies (anti-Lpp20 antibodies) are significantly higher in responders than in non-responders.

In our case, the child's platelet count initially improved following anti-H. pylori therapy, but the subsequent ^13^C-urea breath test suggested persistent infection even 4 weeks after treatment discontinuation, and the child's platelet count decreased accordingly. We speculate that this decrease was indeed linked to the persistent H. pylori infection. It seems to rely on the variation of H. pylori strains. Therefore, the diversity of H. pylori should be emphasized. This includes the individual patient's H. pylori strains present in the stomach and the continuous changes in the composition of H. pylori. Research suggests that platelet count increases in responders within a few weeks of eradication therapy for H. pylori (26). Even in patients without complete eradication of H. pylori, their platelet counts may temporarily increase (26). The mechanism may be that anti-Lpp20 antibodies react with H. pylori Lpp20, but not with platelets; however, Lpp20 can bind to platelets, leading to the immune complex formation and platelet destruction. This reaction may contribute to the transient elevation of platelets (22).

Hashimoto's thyroiditis(HT) is a prevalent cause of hypothyroidism triggered by environmental factors and genetic vulnerability. The disease shows symptoms such as constipation, dry skin, weight gain, intolerance to colds, and fatigue. The diagnosis is based on thyroid function tests that detect high TSH, as we ended TPOAB and/or TGAB. In some instances, the disease may not present any symptoms, referred to as subclinical HT. HT is the most common autoimmune thyroid disease (AITD) and the leading cause of hypothyroidism in iodine-rich regions of the world (27). About 20–30 percent of patients have HT, which is thought to be the result of a combination of genetic susceptibility and environmental factors leading to a loss of immune tolerance, leading to an autoimmune attack on thyroid tissue and disease flares (27). Although ITP as a complication of thyroid disease is rare, it has been reported to be associated with autoimmune thyroid disease, hyperthyroidism, and hypothyroidism (27–31). Treatment for thyroid disease has been shown to improve thrombocytopenia (10, 30–32). Patients with ITP often have autoimmune markers, including TGAB and TPOAB (33). TPOAB has been reported in 31 percent of patients with ITP, and TGAB is associated with lower remission rates (34). The prevalence of antithyroid antibodies in pediatric patients with ITP (11.6%) was higher than in the general pediatric population (1.3%) (34). Thyroid dysfunction is prevalent in patients with ITP, with no significant differences in platelet counts, autoimmune markers other than anti-nuclear antibodies, or clinical diagnosis among patients with and without thyroid disease, and treatment of combined thyroid disease appears to improve platelet counts (28). A study has shown that patients with HT and ITP have recovered their platelet counts after thyroxine supplementation (35). Another case report found a patient with chronic and severe refractory thrombocytopenia who also had hypothyroidism, which responded to thyroxine supplementation, with a restoration of platelet count to the normal range (9).

AITD is a group of conditions where the body's immune system attacks the thyroid gland, resulting in conditions such as Graves' disease (GD), HT, postpartum thyroiditis, and others. These conditions are caused by the infiltration of local lymphocytes T and B cells, activation of apoptotic gene ligands (Fas-L), the release of cytokines, and inflammation and injury of cells and tissues (36). The cytotoxic effects of TPOAB and TGAB can also cause damage and apoptosis of thyroid cells. While genetic and environmental factors can contribute to the development of AITD, there is evidence suggesting that H. pylori infection may be a causative agent (36). Although the link between H. pylori and AITD is not yet conclusive, some studies suggest that H. pylori may be involved in the onset and progression of AITD (37, 38). Large cross-sectional studies have also confirmed that H. pylori can influence the disease progression of AITD and that patients with AITD are more susceptible to H. pylori infection (39). Moreover, a meta-analysis has shown that the prevalence of H. pylori is higher in patients with AITD compared to those without AITD and that the elimination of H. pylori infection can successfully reduce associated auto-antibodies (40). However, other studies suggest that the association between H. pylori and AITD may be more complex, as only patients infected with CagA-positive strains have been linked with AITD (41). In contrast, a randomized controlled trial has shown that H. pylori are not associated with HT (42). Despite the lack of consensus in these studies, the above meta-analysis indicates that H. pylori infection is associated with ITP, HT, and other autoimmune disorders. In our case, the child had decreased TGAB and TPOAB titers on retest after anti-H. pylori and thyroxine supplementation therapy, which seem to be related, but there are confounding factors that need to be verified by further case-control trials.

## 4. Conclusion

Therefore, we emphasize that early screening for H. pylori and thyroid function should be performed in children with ITP who have recurrently reduced platelet counts. Timely detection and eradication of causative factors can be helpful in the treatment and prognosis of children with ITP. Previous literature has found a seeming association between ITP, HT, and H. pylori, but the theory and mechanism are unclear and need to be further confirmed by extensive studies.

## Data Availability

The raw data supporting the conclusions of this article will be made available by the authors, without undue reservation.
